# Comparison of Hypomanic Symptoms Between Bipolar I and Bipolar II Disorders: A Network Perspective

**DOI:** 10.3389/fpsyt.2022.881414

**Published:** 2022-05-12

**Authors:** Wei Bai, Yuan Feng, Sha Sha, Qinge Zhang, Teris Cheung, Dexing Zhang, Zhaohui Su, Chee H. Ng, Yu-Tao Xiang

**Affiliations:** ^1^Unit of Psychiatry, Department of Public Health and Medicinal Administration, Faculty of Health Sciences, Institute of Translational Medicine, University of Macau, Macao, Macao SAR, China; ^2^Center for Cognition and Brain Sciences, University of Macau, Macao, Macao SAR, China; ^3^Institute of Advanced Studies in Humanities and Social Sciences, University of Macau, Macao, Macao SAR, China; ^4^The National Clinical Research Center for Mental Disorders & Beijing Key Laboratory of Mental Disorders Beijing Anding Hospital & The Advanced Innovation Center for Human Brain Protection, School of Mental Health, Capital Medical University, Beijing, China; ^5^School of Nursing, The Hong Kong Polytechnic University, Hong Kong, Hong Kong SAR, China; ^6^Faculty of Medicine, Jockey Club School of Public Health and Primary Care, The Chinese University of Hong Kong, Hong Kong, Hong Kong SAR, China; ^7^School of Public Health, Southeast University, Nanjing, China; ^8^Department of Psychiatry, The Melbourne Clinic and St Vincent’s Hospital, University of Melbourne, Melbourne, VIC, Australia

**Keywords:** Chinese, HCL-32, bipolar disorder, network analysis, comparison

## Abstract

**Background:**

Hypomanic symptoms between bipolar-I disorder (BD-I) and bipolar-II disorder (BD-II) are often indistinguishable in clinical practice. This study compared the network structure of hypomanic symptoms between patients with BD-I and BD-II.

**Methods:**

The 32-item Hypomania Checklist (HCL-32) was used to assess hypomanic symptoms. Network model was generated in BD-I and BD-II patients. Centrality index of strength was used to quantify the importance of each symptom in the network. The Network Comparison Test (NCT) was used to assess the differences in hypomanic symptoms between BD-I and BD-II patients.

**Results:**

Altogether, 423 patients with BD (BD-I: 191 and BD-II: 232) were included. The most central symptom was HCL17 “I am more flirtatious and/or am more sexually active” (strength _*BD*–*I*_ = 5.21) and HCL12 “I have more ideas, I am more creative” (strength _*BD*–*II*_ = 6.84) in BD-I and BD-II samples, respectively. The results of NCT showed that four nodes (HCL12 “I have more ideas, I am more creative,” HCL17 “I am more flirtatious and/or am more sexually active,” HCL23 “My thoughts jump from topic to topic,” and HCL31 “I drink more alcohol”) were significantly different between the BD-I and BD-II samples. Two edges (HCL3 “I am more self-confident”–HCL17 “I am more flirtatious and/or am more sexually active,” and HCL10 “I am physically more active (sport, etc.)”–HCL24 “I do things more quickly and/or more easily”) were significantly stronger in BD-I compared to BD-II patients.

**Conclusion:**

The network structure of hypomanic symptoms is different between BD-I and BD-II patients. Interventions targeting the respective central symptoms and edges should be developed for BD-I and BD-II separately.

## Introduction

Bipolar disorder (BD) is a severe chronic mood disorder characterized by recurrent manic/hypomanic episodes with alternating or intertwining depressive episodes (1, 2), affecting around 45 million people worldwide (3). Both BD type I (BD-I) and BD type II (BD-II) are often misdiagnosed as other psychiatric disorders in clinical practice (4). BD-I is characterized by episodes of mania while in BD-II only hypomania is present; however, the frequency and duration of depressive episodes and the chronicity of illness are usually greater in BD-II (5, 6). The misdiagnosis rate of BD could be up to 76.8% with the most common misdiagnoses being depression, followed by schizophrenia, obsessive compulsive disorder, anxiety disorder and personality disorder (7). Deleterious consequences of misdiagnosis of BD may include the delay of appropriate treatment and incorrect prescription of antidepressants instead of mood-stabilizers, which can lead to manic switching, low therapeutic efficacy, and risk of poor prognosis (8). Therefore, early identification and appropriate treatment are important to reduce the risk of poor prognosis (9, 10). Several clinical diagnostic strategies are available including comprehensive clinical diagnostic interviews and self-reported screening tools, such as the 32-item Hypomania Checklist (HCL-32) (11).

The HCL-32 is a widely used self-report scale with thirty-two symptoms of hypomania to distinguish BD from other psychiatric disorders, particularly depression (11). The HCL-32 has been validated in Chinese psychiatric settings, with satisfactory psychometric properties (12). Previous studies, however, found that due to different clinical features, the HCL-32 performed differently in distinguishing between BD-I and BD-II (11–13). It should be noted that all previous studies focused solely on the total or mean score of the HCL-32. However, no clinical studies of BD at symptom level have been published, even though individual BD symptoms usually have different psychoneurological mechanisms and understanding the inter-relationships between individual BD symptoms has the potential to identify treatment targets (14).

Network analysis, which is a novel approach to model psychiatric disorders as dynamic systems, provides an alternative perspective to visualize the complex associations among psychiatric symptoms (15). In network analysis, each node represents a symptom, each edge represents the association between two symptoms, and the thickness of the edge stands for the magnitude of the association after controlling for other nodes in the network (16). Network analysis can identify symptoms that are highly “central” (influential) in the whole network, which are defined as central symptoms with strong connections to other symptoms; the activation/deactivation of central symptoms could influence other symptoms to be activated/deactivated (15, 17). Clinical interventions that target central symptoms are more likely to improve the severity of psychiatric disorders/syndromes (18). In recent years network analyses have been used in different psychiatric disorders such as BD (19), depression (20), schizophrenia (21), eating disorders (22), and obsessive-compulsive disorder (23). However, to date no studies have compared hypomania symptoms between patients with BD-I and BD-II from the network perspective.

To identify the central symptoms that trigger and maintain hypomanic symptoms from a network perspective, this study compared the network structure of hypomanic symptoms between BD-I and BD-II depressed patients.

## Participants and Methods

### Participants

This study was based on a secondary analysis of two separate projects (24, 25) conducted on the validation of the HCL-32 in China. To be eligible, participants needed to meet following criteria: (1) aged between 16 and 65 years; (2) being diagnosed as BD according to the Diagnostic and Statistical Manual of Mental Disorders, Fourth Edition (DSM-IV) (26) or the International Classification of Diseases, Tenth Revision (ICD-10) (27) based on medical records review; (3) type of BD can be confirmed (BD-I/BD-II); (4) being able to understand the content of the interview. Patients who had a history or ongoing major chronic medical or neurological condition(s), depressive disorders secondary to chronic medical or neurological condition, or received electroconvulsive therapy in the past month were excluded. The study protocol was approved by the Clinical Research Ethics Committee of the respective study hospitals/units and written informed consent was obtained from the participants or their guardians for those who aged younger than 18 years old.

### Measures

A data-collection form was used to collect the participants’ basic socio-demographic and clinical data, including age, gender, age of onset, and the type of BD (BD-I/BD-II).

The HCL-32 (11) is a self-reported questionnaire used to identify hypomanic symptoms in depressed patients. The HCL-32 consists of 32 hypomanic items with each rated yes or no, and participants are asked to remember “a period when you were in ‘high’ state” to indicate whether specific emotions, thoughts, or behaviors at that time (e.g., low-threshold symptoms such as “I wear more colorful and more extravagant clothes/make-up” and “I make more jokes or puns when I am talking,” or high-threshold symptoms such as “I tend to drive faster or take more risks when driving” and “I am more flirtatious and/or am more sexually active”). The Chinese version of HCL-32 has been validated in Chinese population (12) and was used in this study.

### Statistical Analysis

All analyses were conducted using the R program (28). Considering that HCL-32 items were dichotomous variables, the Ising model was adopted (29) to estimate the network structure of hypomanic symptoms in BD-I and BD-II samples using the function estimateNetwork in package bootnet version 1.4.3 (30). In network parlance, each symptom is shown as a node and the association between two items after controlling for other nodes is presented as an edge. A thicker edge indicates a stronger association, while the green color of the edge represents a positive association, and the red color means a negative association. The package qgraph version 1.6.9 (16) was used to visualize the network.

To quantify the importance of each symptom in the network, centrality indices (e.g., strength, betweenness, and closeness) were used, with a higher value indicating a more influential role in the network. As demonstrated by previous studies (31, 32), results of betweenness and closeness are unreliable; thus, the stable centrality index—strength (i.e., the sum of all edge weights of a given node with all other nodes) was used in this study and computed by the function centralityPlot in package qgraph version 1.6.9 (16). Additionally, predictability, a measure indicating the interconnectedness of a node with its neighboring nodes, was computed by the function predict of the package mgm version 1.2-12 (33). In the layout of the network, the value of predictability was expressed as the area in the ring of each node.

To assess the differences in hypomanic symptoms between bipolar I and bipolar II patients, the Network Comparison Test (NCT) was used in the package NetworkComparisonTest version 2.2.1 (34). The NCT is a permutation test, aiming to investigate differences in the global strength, network structure, each edge weight, as well as individual node strength. Based on previous studies (35, 36), results with and without Bonferroni-Holm correction in NCT were reported.

Finally, to assess the stability of the network, the package bootnet version 1.4.3 (30) was applied with 1,000 bootstraps for each node in the bipolar I and bipolar II. The correlation stability (CS)—coefficient was used, with the value indicating the maximum proportion of cases that could be dropped from the original sample to maintain the correlation (*r* = 0.7) between the subset sample and the original sample. The recommended value of CS-coefficient is above 0.25, and above 0.5 is preferred (30).

## Results

### Characteristics of Participants

In total, 423 patients with BD who met study criteria (265 females and 158 males) participated in the study. The mean age of patients was 35.4 years [standard deviation (*SD*) = 12.2], and the mean age of onset was 27.8 years (*SD* = 11.2). Among them, 191 and 232 patients were diagnosed with BD-I and BD-II, respectively. There were no significant differences in the mean age (*t* = 0.42, *P* = 0.68), mean age of onset (*t* = 0.86, *P* = 0.39), and gender distribution (χ^2^ = 0.46, *P* = 0.50) between patients with BD-I and BD-II. The percentage of each hypomanic symptom in HCL-32 is presented in Table 1.

**TABLE 1:** Descriptive statistics of HCL-32 items in patients with bipolar I/II disorders.

Items	BD-I (*N* = 191)			BD-II (*N* = 232)		
	Percentage*^a^* (%)	Strength*^b^*	Predictability (%)	Percentage*^a^* (%)	Strength*^b^*	Predictability (%)
HCL1	72.3	0	22.2	57.8	0	16.1
HCL2	79.1	2.03	37.8	79.3	1.53	34.0
HCL3	77.5	5.04	46.6	78.4	3.46	41.7
HCL4	69.6	2.51	36.0	68.1	2.22	35.5
HCL5	68.6	4.75	45.5	65.9	2.64	41.0
HCL6	49.7	1.69	36.8	43.1	0.66	26.5
HCL7	29.3	0	26.0	13.4	0.62	15.3
HCL8	49.7	2.69	40.7	44.4	2.30	25.5
HCL9	47.1	3.03	44.2	37.1	1.04	28.0
HCL10	68.1	1.96	37.6	68.1	1.00	21.0
HCL11	73.3	2.35	56.4	69.0	4.43	56.0
HCL12	70.7	3.63	53.1	65.9	6.84	62.6
HCL13	60.7	2.59	41.8	65.5	3.28	47.3
HCL14	51.3	2.35	37.4	57.3	1.06	30.1
HCL15	63.9	4.99	49.0	66.4	3.83	44.3
HCL16	42.9	2.20	43.9	37.5	2.15	43.1
HCL17	45.5	5.21	56.5	39.7	1.40	34.3
HCL18	75.9	2.92	39.6	80.2	3.51	39.6
HCL19	73.8	3.09	44.2	72.4	3.64	48.9
HCL20	56.5	1.69	40.3	58.6	3.32	47.9
HCL21	59.7	2.62	42.7	45.3	0.74	28.8
HCL22	60.2	1.82	43.8	58.2	2.45	40.0
HCL23	59.2	3.80	45.8	50.4	0.00	27.7
HCL24	68.6	3.36	41.1	72.4	2.74	44.2
HCL25	55.0	2.64	44.8	38.8	2.83	37.4
HCL26	50.8	3.70	53.7	31.0	3.50	54.0
HCL27	47.6	3.50	44.4	30.2	2.94	52.5
HCL28	61.3	4.18	51.3	67.7	2.55	42.2
HCL29	21.5	0.85	35.8	11.2	0.00	28.9
HCL30	21.5	3.07	51.8	13.4	2.18	37.8
HCL31	18.3	4.94	64.3	12.9	2.18	38.4
HCL32	23.0	1.74	48.9	14.7	0	18.4

*^a^These two columns presented the percentage of hypomanic symptoms. ^b^The values of strength were shown as raw data. BD, bipolar disorder; HCL, Hypomania Check List.*

### Network Analysis and Comparison

**FIGURE 1:**
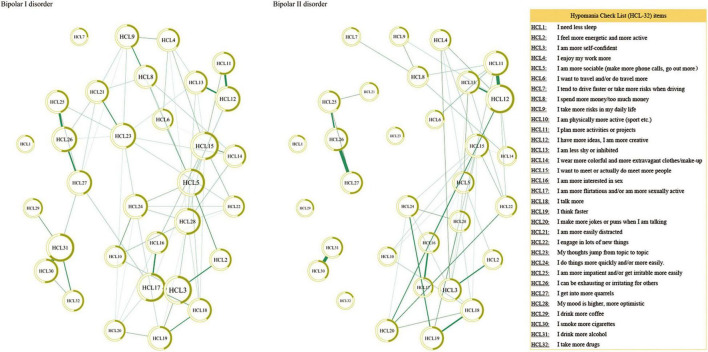
Network structure of hypomanic symptoms in individuals with bipolar I/II disorders.

Figure 1 presents the network structure for the BD-I and BD-II samples. The strongest edge in the BD-I sample was the connection between HCL30 “smoke more cigarettes” and HCL31 “I drink more alcohol,” followed by the edge HCL3 “I am more self-confident”-HCL17 “I am more flirtatious and/or am more sexually active” and the edge HCL11 “I plan more activities or projects”—HCL12 “I have more ideas, I am more creative.” In the BD-II sample, the edge between HCL11 “I plan more activities or projects” and HCL12 HCL12 “I have more ideas, I am more creative” has the strongest association, followed by the edge HCL26 “I can be exhausting or irritating for others”—HCL27 “I get into more quarrels,” and HCL30 “smoke more cigarettes”—HCL31 “I drink more alcohol.” The NCT results found that there was no significant difference in global strength (BD-I sample: 45.47; BD-II sample: 35.53; *S* = 9.94, *P* = 0.25), but there was significant difference in the network structure (*M* = 2.04, *P* = 0.02) between the two samples. Then each edge was further compared, and Table 2 presents the significantly different edges between both samples. Two edges (HCL3 “I am more self-confident”—HCL17 “I am more flirtatious and/or am more sexually active,” and HCL10 “I am physically more active (sport, etc.)”—HCL24 “I do things more quickly and/or more easily”) remained significantly different between both samples after Bonferroni-Holm correction.

**TABLE 2:** Edge differences between BD-I and BD-II samples.

Variable 1		Variable 2		*P*-value
HCL3	I am more self-confident	HCL17	I am more flirtatious and/or am more sexually active	<0.001*
HCL3	I am more self-confident	HCL13	I am less shy or inhibited	0.041
HCL4	I enjoy my work more	HCL12	I have more ideas, I am more creative	0.019
HCL4	I enjoy my work more	HCL9	I take more risks in my daily life	0.026
HCL5	I am more sociable	HCL8	I spend more money/too much money	0.019
HCL5	I am more sociable	HCL20	I make more jokes or puns when I am talking	0.021
HCL5	I am more sociable	HCL9	I take more risks in my daily life	0.031
HCL9	I take more risks in my daily life	HCL21	I am more easily distracted	0.031
HCL10	I am physically more active (sport, etc.)	HCL24	I do things more quickly and/or more easily.	<0.001*
HCL11	I plan more activities or projects	HCL19	I think faster	0.022
HCL12	I have more ideas, I am more creative	HCL20	I make more jokes or puns when I am talking	0.014
HCL13	I am less shy or inhibited	HCL22	I engage in lots of new things	0.006
HCL17	I am more flirtatious and/or am more sexually active	HCL27	I get into more quarrels	0.019
HCL20	I make more jokes or puns when I am talking	HCL22	I engage in lots of new things	0.043
HCL23	My thoughts jump from topic to topic	HCL24	I do things more quickly and/or more easily.	0.005

*Remained significant after Bonferroni correction. BD, bipolar disorder; HCL, Hypomania Check List.

**FIGURE 2:**
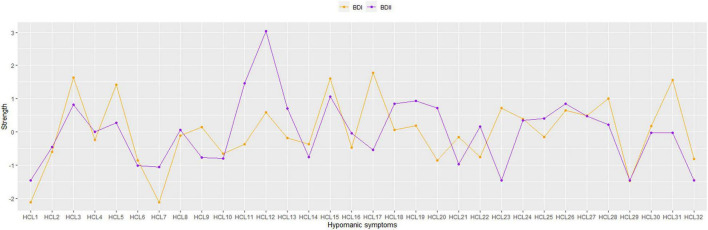
Strength centrality for each node included in the network (z-scores).

As shown in Table 1, HCL31 “I drink more alcohol” has the highest predictability in the BD-I sample and the mean predictability was 43.9%, suggesting that an average of 43.9% variance could be explained by each node’s neighboring nodes in the BD-I sample. The mean predictability was 37.2% in the BD-II sample, with HCL11 “I plan more activities or projects” having the highest predictability. In term of node strength, HCL17 “I am more flirtatious and/or am more sexually active” had the highest value in the BD-I sample, followed by HCL3 “I am more self-confident” and HCL15 “I want to meet or actually do meet more people” (Figure 2). In the BD-II sample, the most central symptom was HCL12 “I have more ideas, I am more creative,” followed by HCL11 “I plan more activities or projects” and HCL15 “I want to meet or actually do meet more people” (Figure 2). Then the strength of each node was further compared by NCT, and results showed that there were significant strength centrality differences in HCL12 “I have more ideas, I am more creative” (*P* = 0.029), HCL17 “I am more flirtatious and/or am more sexually active” (*P* = 0.005), HCL23 “My thoughts jump from topic to topic” (*P* = 0.005), and HCL31 “I drink more alcohol” (*P* = 0.035) between the BD-I and BD-II samples. However, these differences disappeared after Bonferroni-Holm correction was adopted.

### Network Stability

**FIGURE 3:**
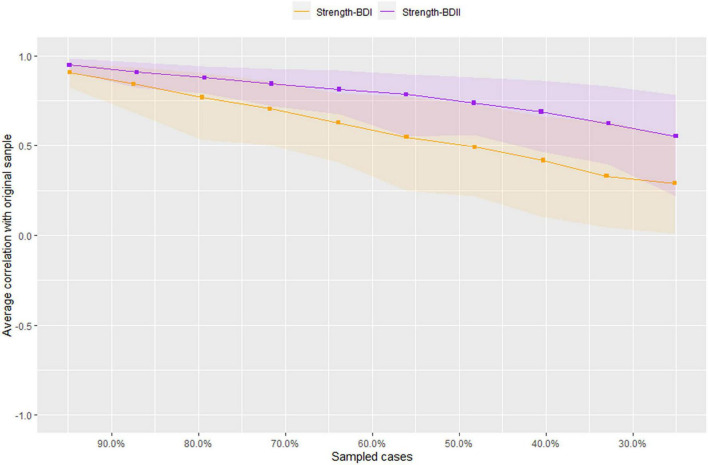
Stability of network structures in patients with bipolar I/II disorders. The x-axis represents the included percentage of study samples, and the y-axis indicates the correlations between the original centrality index (i.e., strength) and the estimated one after dropping different proportion of study samples. Different color lines indicate different network properties (CS-C _*BD*–*I*_ = 0.126, CS-C _*BD*–*II*_ = 0.362).

Figure 3 illustrates the results of robustness analyses in both BD-I and BD-II samples. The results show an acceptable stability for BD-II sample with a value of 0.362 for strength CS-coefficient. For BD-I sample, however, the CS-coefficient was 0.126, which was below the recommended 0.25, indicating that caution should be taken when interpreting this measure.

## Discussion

This was the first study that compared the network structure of hypomanic symptoms between patients with BD-I and BD-II. As indicated in previous studies (15, 18, 31), high centrality of a symptom suggests the strong association with other symptoms in a network, and can play an important role in triggering the occurrence and maintaining the psychopathology model of a psychiatric disorder. The central symptoms can also be intervention targets in treating the disorder. While a positive edge between two symptoms represents the co-occurrence between them and a strong edge indicates that both symptoms may be strongly connected, targeting either of them can improve the other symptom (15, 37).

We found that the most central symptom was HCL17 “I am more flirtatious and/or am more sexually active” in the BD-I sample, while the most central symptom was HCL12 “I have more ideas, I am more creative” in the BD-II sample. The strength indices of two nodes showed significant differences between both samples (HCL17: BD-I node strength = 5.21, BD-II node strength = 1.40, *P* = 0.005; HCL-12: BD-I node strength = 3.63, BD-II node strength = 6.84, *P* = 0.029). This finding was consistent with the notion that BD-I patients usually experience more severe manic/hypomanic episodes than BD-II patients (2). A previous study also found that compared to BD-II patients, BD-I patients often had increased implicit sexual interest (38), while another study found higher hypersexuality in BD-I patients and elevated mood in BD-II patients (39), which are consistent with our findings. Neuroimaging studies found that compared to BD-I patients, those with BD-II had decreased surface area in the right long insula (40), while neurophysiological research showed higher ratios of P50 (i.e., an event to measure sensory gating) cerebral potential in BD I patients than those in BD II patients (41), both of which may be associated with different clinical symptoms between BD-I and BD-II patients. Additionally, network comparison test for node strength between BD-I and BD-II samples revealed that the strength indices of HCL23 “My thoughts jump from topic to topic” (BD-I node strength = 3.80, BD-II node strength = 0.00, *P* = 0.005) and HCL31 “I drink more alcohol” (BD-I node strength = 4.94, BD-II node strength = 2.18, *P* = 0.035) were significantly higher in BD-I compared to BD-II samples, indicating that they may be potential treatment targets to improve manic/hypomanic symptoms in BD-I patients.

HCL3 “I am more self-confident” was another central symptom in BD-I patients, but there was no significant difference in its node strength between BD-I and BD-II patients, which partly supports a previous finding that the symptom “Lack of social self-confidence” was not obvious in BD-II compared to BD-I patients, and the difference did not reach significance level (42). We found that HCL11 “I plan more activities or projects” was a central symptom in BD-II patients, but there was no significant difference in its node strength different between both samples. Though the symptom HCL11 “I plan more activities or projects” is more common in BD-I patients (43), our network analysis revealed that it may be the backbone of hypomanic symptom network in BD-II patients. Compared to traditional statistical approach, network analysis could identify influential symptoms after controlling for other hypomanic symptoms. The symptom HCL15 “I want to meet or actually do meet more people” was another central node in both BD-I and BD-II samples, without significant group difference, indicating it could be targeted in the treatment of both BD subtypes.

In terms of edge weight, we found that two edges HCL30 “smoke more cigarettes”—HCL31 “I drink more alcohol,” and HCL11 “I plan more activities or projects”—HCL12 “I have more ideas, I am more creative” showed strongly positive connections in both samples, indicating that the two edges were strong and stable, and the two symptoms included in each edge tended to co-occur in BD patients. The strong association between cigarette consumption and smoking was found in the general population (44, 45) and in psychiatric patients (46, 47). For example, compared to non-smokers, current smokers and ex-smokers had 13.5- and 12.1-times higher risk to engage in hazardous alcohol use, respectively, among psychiatric outpatients (47). The common genetic factors (48) and the mutually enhancing effects of alcohol and nicotine (49) may contribute to the co-occurrence of alcohol and nicotine addiction. The link between HCL11 “I plan more activities or projects”—HCL12 “I have more ideas, I am more creative” may be explained by the presence of increased creativity in many BD patients. A meta-analysis found an increased creativity in BD patients (50), while another study also found that persons with BD tended to have higher level of creative accomplishment compared with those without BD (51).

We found that the edge HCL26 “I can be exhausting or irritating for others”—HCL27 “I get into more quarrels” was strong in BD-II patients, which could be explained by a previous finding that interpersonal tension with close others was linked to being irritable in BD-II patients (52). A strong edge (HCL3 “I am more self-confident”—HCL17 “I am more flirtatious and/or am more sexually active”) was found in BD-I patients, which was significantly stronger than that in BD-II patients. The association between “much more sex” and “self-confidence” in individuals with BD has been reported previously (53). Compared to BD-II, another significantly stronger edge in BD-I sample was HCL10 “I am physically more active (sport, etc.)”—HCL24 “I do things more quickly and/or more easily.” The notion that BD-I patients usually experience more severe symptoms during the manic episode than BD-II patients (2) could partly explain these stronger edges (i.e., HCL3–HCL17, and HCL10–HCL24) in BD-I patients.

The strength of this study was the use of network approach to compare hypomanic symptoms between BD-I and BD-II patients. However, several limitations should be noted. First, the stability test index CS-coefficient in BD-I was lower than recommended (30), which may be caused by the small sample size. Second, this study was conducted in China, therefore, the generalizability of the findings could not be applied to other regions. Third, hypomanic symptoms were only assessed with the HCL-32 in this study and therefore the findings need to be confirmed in future studies using other measures of BD. Finally, the HCL-32 is a self-reported questionnaire, hence the possibility of recall bias could not be excluded.

In conclusion, the symptom structure of hypomanic network is different between patients with BD-I and BD-II. Interventions targeting the respective central symptoms and edges should be developed for BD-I and BD-II separately.

## Data Availability Statement

The Clinical Research Ethics Committees of the respective study hospitals/units that approved the study prohibit the authors from making the research dataset of clinical studies publicly available. Readers and all interested researchers may contact Y-TX (xyutly@gmail.com) for details. Y-TX will apply to the Clinical Research Ethics Committees of the respective study hospitals/units for the release of the data.

## Ethics Statement

The studies involving human participants were reviewed and approved by the Medical Ethics Committee of the First Affiliated Hospital of Zhengzhou University. Written informed consent to participate in this study was provided by the participants’ legal guardian/next of kin.

## Author Contributions

WB completed the data collection, analysis, interpretation, and drafted the manuscript. SS completed the study design. YF, QZ, TC, DZ, and ZS completed the data collection, analysis, and interpretation. Y-TX completed study design and drafted the manuscript. CN completed the critical revision of the manuscript. All the authors finished the approval of the final version for publication.

## Conflict of Interest

The authors declare that the research was conducted in the absence of any commercial or financial relationships that could be construed as a potential conflict of interest. The reviewer JL declared a shared affiliation with the author TC at the time of review.

## Publisher’s Note

All claims expressed in this article are solely those of the authors and do not necessarily represent those of their affiliated organizations, or those of the publisher, the editors and the reviewers. Any product that may be evaluated in this article, or claim that may be made by its manufacturer, is not guaranteed or endorsed by the publisher.
